# TCMGIS-II based prediction of medicinal plant distribution for conservation planning: a case study of *Rheum tanguticum*

**DOI:** 10.1186/1749-8546-5-31

**Published:** 2010-08-25

**Authors:** Hua Yu, Caixiang Xie, Jingyuan Song, Yingqun Zhou, Shilin Chen

**Affiliations:** 1 Institute of Medicinal Plant Development, Chinese Academy of Medical Sciences, Peking Union Medical College, Beijing 100193, China; 2 Hubei University of Chinese Medicine, Wuhan 430065, China; 3 Technology Development Centre, China National Group Corporation of Traditional and Herbal Medicine, Beijing 100094, China

## Abstract

**Background:**

Many medicinal plants are increasingly endangered due to overexploitation and habitat destruction. To provide reliable references for conservation planning and regional management, this study focuses on large-scale distribution prediction of *Rheum tanguticum *Maxim. ex Balf (*Dahuang*).

**Methods:**

Native habitats were determined by specimen examination. An improved version of GIS-based program for the distribution prediction of traditional Chinese medicine (TCMGIS-II) was employed to integrate national geographic, climate and soil type databases of China. Grid-based distance analysis of climate factors was based on the Mikowski distance and the analysis of soil types was based on grade division. The database of resource survey was employed to assess the reliability of prediction result.

**Results:**

A total of 660 counties of 17 provinces in China, covering a land area of 3.63 × 10^6 ^km^2^, shared similar ecological factors with those of native habitats appropriate for *R. tanguticum *growth.

**Conclusion:**

TCMGIS-II modeling found the potential habitats of target medicinal plants for their conservation planning. This technology is useful in conservation planning and regional management of medicinal plant resources.

## Background

More than one-tenth of plant species are used in drugs and health products [1]. The demand for herbal drugs and health products is steadily growing [2]. Thus, many medicinal herbs are threatened by overexploitation, habitat destruction and lack of proper cultivation practices. Some wild species are disappearing at alarming rates [3,4]. *Rheum tanguticum *Maxim. ex Balf (*Dahuang*) is one of those species. *R. tanguticum *belongs to the family Polygonaceae and is a high-altitude perennial herb sensitive to high temperature, mainly found in the alpine regions of temperate and subtropical Asia, especially in Southwest and Northwest China (e.g. Sichuan, Gansu and Qinghai) [5,6]. As a source for rhubarb according to the *Chinese Pharmacopoeia *and a purgative and anti-inflammatory agent [7], *R. tanguticum *has been overexploited, suffering from replant diseases, inadequate seed dispersal, low reproductive efficiency and narrow distribution and habitat fragmentation, leading to its declines in the wild resources [6,8].

*In-situ *conservation, which considered as the method of conserving endangered species in their wild habitats, is promising in protecting indigenous species and maintaining natural communities along with their intricate network of relationships [9]. As habitat degradation and destruction is increasing, *ex-situ *conservation regarded as the process of cultivating and naturalizing endangered species outside of their original habitats, has become a practical alternative [10-12], especially for those overexploited and endangered medicinal plants with slow growth, small abundance and replant diseases [10,13], e.g. *Paris *species in family Trilliaceae and *Panax *species in family Araliaceae [14]. *Ex-situ *cultivation becomes an immediate action to sustain medicinal plant resources [11,12].

Understanding the geographical distribution of plant species is essential for their *ex-situ *conservation activities [1,15]. Although many plant species can be successfully introduced, cultivated and naturalized in a wide range of habitats across countries and continents [16], their growth and distribution in different habitats are based on local indicators [17], e.g. soil properties, climate conditions and environmental features [18]. Aguilar-Stoen and Moe (2007) found that many medicinal plants thriving in harsh habitats and disturbed areas are of high medicinal efficacy because rocky and dry habitats stimulate their secondary metabolites [19]. Many plants are only found in places where the habitat is congruent with their growth [18], e.g. the propagation and quality of *Banksia serrata *varied among habitats [20]. Variations in growth and metabolites of medicinal plants among niches make *ex-situ *conservation habitat-specific.

Geographical prediction of plant distribution is important to resource conservation planning and regional management decisions [21]. Geographic Information System (GIS) is useful in predicting the spatial distribution of target species [22]. GIS assesses multiple interdependent abiotic factors, e.g. solar radiation, air temperature, precipitation and soil properties [23], affecting plant distribution, models the environmental niches of target plants [24] and refines their distribution maps for conservation planning [25].

A GIS-based computer program (TCMGIS-I) was developed specially for the distribution prediction of Chinese medicine (CM) [25,26]. Integrating national geographic, climate and soil type databases of China, TCMGIS-I was able to determine the impacts of environmental gradients and predict the large-scale distribution of target medicinal plants [26]. Tests with some common medicinal plants (e.g. *Panax ginseng*, *Panax quinquefolium*, *Glycyrrhiza uralensis *and *Artemisia annua*) demonstrated that TCMGIS-I prediction was consistent with the actual plants' distribution patterns [27-30].

While TCMGIS-I captures data from literature, TCMGIS-II can perform more precise variable extraction from the native habitats of target medicinal plants. Factors such as elevation, air temperature, solar radiation, precipitation and soil properties are considered by TCMGIS-II. Moreover, TCMGIS-II defines the native habitats of a target plant through specimen examination and extracts the target variables of native habitats from its databases.

The present study aims to determine (1) the most important ecological factor(s) on the distribution of *R. tanguticum*, (2) whether the prediction results are consistent with survey data and (3) the implications of the prediction results for the conservation planning of *R. tanguticum*.

## Methods

### Database descriptions

Based on a spatially referenced GIS model, TCMGIS-II integrated four databases, including the national geographic, climate and soil type databases of China which were used to generate distribution models and the database of resource survey which was used to assess the quality of a model.

The geographic database of China was a digital chart (scale 1:1,000,000) at national, provincial, regional and county levels, including a series of vector maps of layers, i.e. manuals on roads, contours, geology and administrative boundaries, with all points covered with a geographic coordinate system (e.g. latitude, longitude and elevation).

The climate database of China was derived from the national climate data coving from the period of 1971 to 2000 extracted from the climate records of the state meteorological administration of China. The database included climate attributes related to plant growth, e.g. sunshine duration, relative humidity, annual precipitation, accumulated temperature, mean annual temperature, mean March temperature, annual maximum/minimum temperature and annual mean maximum/minimum temperature. The climate data were available in GIS along with data of latitude, longitude and elevation.

The soil type database of China covered a total of 2,444 counties, containing a series of vector soil maps (scale 1:1,000,000) and soil attributes and mapping unit boundaries. The soil data were classified into 12 orders, 29 suborders, 61 groups, 235 subgroups and 909 families as the basic elements of the map layers [31].

The database of resource survey was generated with the third national resource survey of CM in China, covering a total of 11,118 plant species in 2312 genera of 385 families, including 298 fungi, 114 algae, 43 mosses, 55 lichens, 455 ferns, 126 gymnosperms and 10,027 angiosperms [32], as well as descriptions on the abundance and distribution patterns of 138 rare and endangered medicinal plants, 126 of which were converted into digital charts (scale 1:1,000,000).

### Model descriptions

TCMGIS-II identified, analyzed and displayed geographically referenced information, using two major data models (i.e. raster and vector). Raster model in 1.0 × 1.0 km^2 ^grids detected the grids sharing similar ecological factors with those of the native habitats of a target medicinal plant. Vector model stacked the layers of those factors to determine the distribution areas and ranges.

### Extraction of ecological factors from native habitats

**Figure 1:**
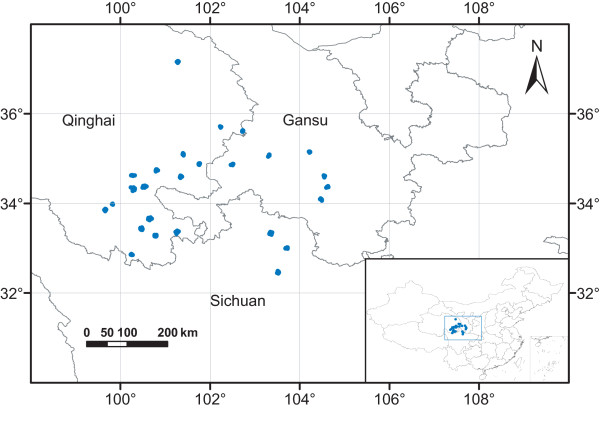
**Native habitats of *Rheum tanguticum *Maxim. ex Balf Blue plotsin 26 towns were set up for the extraction of target variables.**

Based on 75 type specimens of wild *R. tanguticum *from Chinese Virtual Herbarium, we set up 206 plots in 26 towns of nine counties in the provinces of Gansu, Qinghai and Sichuan (Figure 1), the native habitats of *R. tanguticum*. The ecological factors of the plots were extracted by TCMGIS-II, including elevation, soil type, sunshine duration, relative humidity, annual precipitation, accumulated temperature, mean annual temperature, mean March temperature, annual maximum/minimum temperature and annual mean maximum/minimum temperature (Table 1). The variables extracted from the native habitats were set as target variables for distance analysis with grids.

**Table 1:** Variables extracted from the native habitats of *Rheum tanguticum *Maxim. ex Balf based on TCMGIS-II combined geographic, climate and soil type databases

Variable	Unit	Range	Mean ± SE	*F*-value	***C***_***v***_**(%)**
Elevation	m	1980, 4550	3630 ± 44	191.2***	17.4
Relative humidity	%	54.8, 69.0	63.7 ± 2.2	219.3***	49.6
Sunshine duration	hr/yr	1897, 2704	2450 ± 13	301.7***	7.6
Annual precipitation	mm	331, 839	574 ± 7	233.2***	17.5
Accumulated temperature	°C	3193, 22451	9517 ± 951	277.1***	143.4
Mean annual temperature	°C	5.1, 13.1	8.6 ± 0.1	92.6***	16.7
Mean March temperature	°C	-8.0, -2.0	-4.5 ± 0.2	42.3***	63.8
Minimum temperature	°C	-24.8, -10.6	-19.1 ± 0.2	165.8***	15.0
Maximum temperature	°C	12.9, 24.4	17.2 ± 0.2	119.5***	16.7
Mean minimum temperature	°C	-15.6, -5.1	-11.2 ± 0.2	129.8***	25.6
Mean maximum temperature	°C	6.0, 18.2	10.4 ± 0.2	103.3***	27.6
Soil type*	pH	5.9, 8.5	6.8 ± 0.1	112.4***	21.1

* Soil type was assigned according to soil grade division in TCMGIS-II program.

Values of pH were employed as an indicator of soil types for statistical analysis.

*F*-value indicates the difference in target variable extracted from different native habitats (*** *P *< 0.001, ** *P *< 0.01, and * *P *< 0.05).

SE: standard error of means

*C*_*v*_: coefficient of variation

### Data normalization and distance analysis

As there were variations in factors (e.g. climate factors and soil type), TCMGIS-II normalized data by joining the mean absolute deviation of each pair of factors. To determine the similarity rate between grids and target variables from native habitats, we conducted distance measurement based on grid-based analysis. Distance analysis of soil was conducted according to grade division, while the distance analysis of elevation and climate factors was conducted based on Mikowski distance [33], in TCMGIS-II as follows:

$$d_{ij}(q)={\left(\sum_{i=1}^{n}{\left|x_{ij}-y_{ij}\right|}^{q}\right)}^{1/q}$$

Where *x*_*ij *_is the grid value and *y*_*ij *_is a target variable.

When *q *= 1, it is Manhattan distance.

When *q *= 2, it is Euclidean distance.

Long distance indicates low similarity rates while short distance indicates high similarity rates.

### Spatial distribution division and model quality assessment

Division on spatial distribution of *R. tanguticum *was established according to the grid-based clustering. The areas sharing similar ecological factors with those of native habitats were favorable for *R. tanguticum *distribution. The spatially predicted areas were divided into three types, namely the favorable (with similarity rate ≥95%), suitable (with similarity rate 90-95%), and slightly appropriate (with similarity rate < 90%) for *R. tanguticum *distribution.

To assess the reliability of the spatial prediction on *R. tanguticum *distribution, we employed the database of resource survey as a measure. The overlapping part between distribution range predicted by TCMGIS-II and that recorded by resource survey indicates the congruency, the part with prediction result without survey data suggests the potential distribution of *R. tanguticum*, and the rest part with survey data beyond prediction result indicates the contradiction between prediction result and survey data.

### Statistical analyses

To detect the variations in the abiotic factors (e.g. elevation, air temperature, solar radiation, precipitation and soil properties in Table 1) of different native habitats, we employed the coefficient of variation (*C*_*v*_) as a measure [34]. It is defined as the follows:

$$C_{v}=\frac{\sigma }{\mu }\times 100\%$$

Where σ is the standard deviation and μ is the mean.

**Figure 2:**
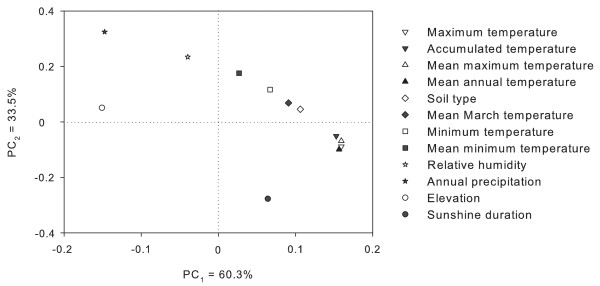
**Plot of component scores determined by principal component analysis on target variables from the native habitats of *Rheum tanguticum *Maxim. ex Balf PC indicates a principal component.**

We employed one-way analysis of variance (one-way ANOVA) to analyze the differences in the abiotic factors responding to different native habitats (Table 1), and principal components analysis (PCA) to evaluate the contributions of the abiotic factors to *R. tanguticum *distribution (Figure 2).

## Results

### Target variables extracted from native habitats

TCMGIS-II extracted the target variables from 206 plots in the native habitats of *R. tanguticum *(Figure 1, Table 1). The results showed that the target variables varied significantly among different native habitats (Table 1, *P *< 0.001), with coefficient of variation ranging from 7.6% in sunshine duration to 143.4% in accumulated temperature, and the native habitats exhibited high elevation and abundant sunshine with moderate cool and dry climate in mild acid and basic soils (Table 1). Using PCA, we extracted two principal components (PCs) which accounted for 93.8% of the contribution of target variables in terms of *R. tanguticum *distribution (Figure 2). The PC_1 _(PC_1 _= 60.3%) was mainly related to temperatures (e.g. annual maximum, annual mean maximum, mean annual and acuminated temperatures) and the PC_2 _(PC_2 _= 33.5%) was mainly contributed by annual precipitation and relative humidity. However, elevation and annual precipitation were negatively correlated to PC_1_, and sunshine duration was negatively contributed to PC_2 _(Figure 2).

### Prediction result of potential distributions

**Figure 3:**
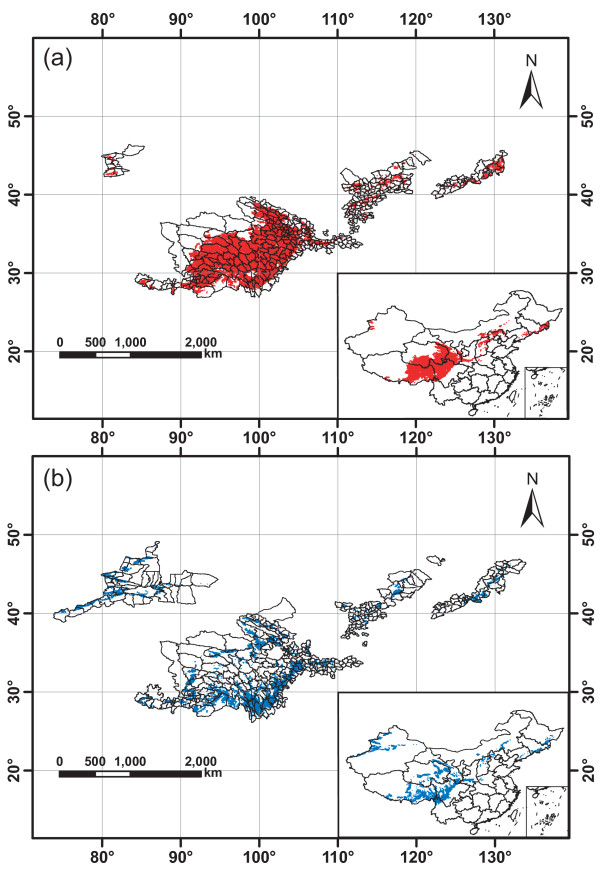
**Spatial distribution of *Rheum tanguticum *Maxim. ex Balf predicted by TCMGIS-II.** (a) Favorable area with similarity rate ≥95% and (b) suitable area with similarity rate 90-95%. Longitude (°E) and latitude (°N) are given.

**Figure 4:**
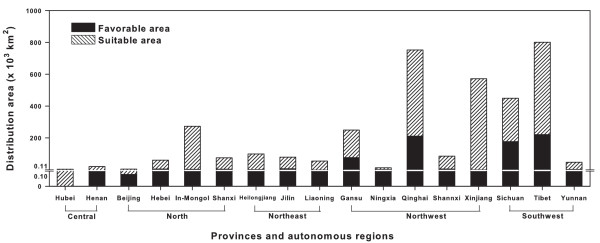
**Detailed distribution of *Rheum tanguticum *Maxim. ex Balf predicted by TCMGIS-II in China.** Favorable area with similarity rate ≥95% (dark) and the suitable area with similarity rate 90-95% (hatched).

The spatial distribution of *R. tanguticum *was established by overlapping the layers of those geographic, climate and soil factors based on distance analyses. The scope of favorable areas (with similarity rate ≥95%) was within 80°26'-131°21'E and 27°03'-45°21'N (Figure 3a), covering 395 counties in 13 provinces such as Xizang (Tibet), Sichuan, Qinghai and Gansu in China with a land area of 7.46 × 10^5 ^km^2 ^(Figure 4). The scope of suitable areas (similarity rate 90-95%) was within 74°05'-132°24'E and 26°38'-47°22'N (Figure 3b), covering 396 counties in 17 provinces with a land area of 2.89 × 10^6 ^km^2 ^(Figure 4). In addition to 131 counties of both favorable and suitable ranges, 660 counties were tested suitable for *R. tanguticum *cultivation (similarity rate ≥90%).

### Comparison between prediction results and survey data

**Figure 5:**
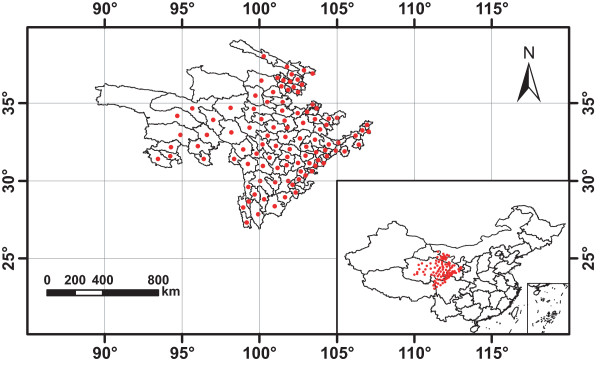
**Distribution map of rhubarb generated based on the database of resource survey. **The red dots show that there existed the wild resources of *R. tanguticum *in the counties. Longitude (°E) and latitude (°N) are given.

**Figure 6:**
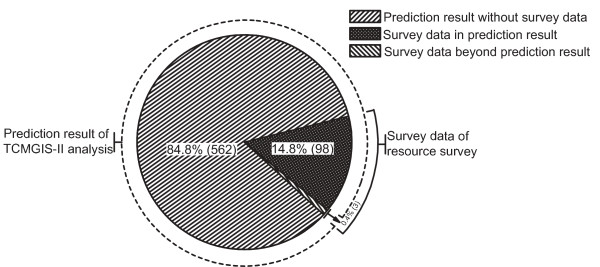
**Comparison between the distribution counties of *Rheum tanguticum *Maxim. ex Balfpredicted by TCMGIS-II and recorded by the survey data.** Latticed: the counties of survey data in prediction result. Left hatched: those of prediction results without survey data. Right hatched: those of survey data beyond prediction results. The percentage and number of counties in each part are given.

Rhubarb distributed in 101 counties in Sichuan, Xizang and Qinghai provinces within the range of 89°25'-107°16'E and 27°05'-39°06'N (Figure 5). Comparison between the distribution counties predicted by TCMGIS-II modeling and recorded by resource survey demonstrated the high quality of prediction result (Figure 6). Specifically, a total of 663 counties were listed by the survey data and prediction result, with 97.0% of survey data covered by the prediction result of TCMGIS-II analysis. The majority (85.2%) of prediction data corresponded to no survey data and 2.9% of survey data did not overlap with the prediction results.

## Discussion

The ecological factors from native habitats suggest that *R. tanguticum *grows at high plateau (e.g. alpine meadow, grassland and shrub) with cool climate, abundant sunshine, moderate precipitation and basic soils (e.g. humus-rich loam and sandy loam) and that its distribution is mainly influenced by temperature (e.g. annual maximum, mean annual and acuminated temperatures), annual precipitation and relative humidity. The prediction results by TCMGIS-II confirmed the distribution data.

Many plant species have evolved to be habitat-specific and sensitive to environmental conditions [35], and those growing at the sites congruent with their native habitats are the most potent [17]. For example, *R. tanguticum *from Gansu and Qinghai is recorded as a source of rhubarb in the *Chinese Pharmacopoeia *due to its high potency [7,32]. The present study found that a large portion of predictive distributions were beyond what survey data covered (e.g. Xinjiang, Inner-Mongolia and Shanxi provinces), agreeing with the notion that prediction of distribution may help locate habitats for conservation [24,36], giving insights into the discovery of potential habitats for *R. tanguticum *cultivation.

Interestingly, a small portion of survey data does not overlap with prediction result, e.g. Muli in Sichuan and Zhongdian in Yunnan. According to the *Chinese Pharmacopoeia*, there are three prescribed sources (i.e. *R. tanguticum*, *R. palmatum *and *R. officinale*) for rhubarb [7]. The survey data cover the three *Rheum *species. On the other hand, the databases of TCMGIS-II include many abiotic factors (e.g. topographic features, climate conditions and soil properties) but not the effects of dynamic biotic interactions and species-specific features on a large scale. Many plant species are sensitive to both abiotic and biotic factors, such as competitor plants and symbiotic species [37,38].

In the present study, the distribution of *R. tanguticum *predicted by TCMGIS-II program was confirmed by the resource survey data. We expect that the TCMGIS-II modeling is useful in conservation planning and regional management for the threatened medicinal plants [19]. Both conservation and sustainable utilization of medicinal plants require robust large-scale assessment of their distribution and regionalization [1]. Lack of data and limit of model validity are barriers for the studies on distribution of medicinal plants on a large scale [39]. Thus, more data and model verification are necessary for further studies and GIS developments.

## Conclusion

TCMGIS-II program was confirmed to be useful in the discovery of potential habitats congruent with the native habitats of target medicinal plants. This technology provides reliable references for the conservation planning and regional management of endangered and threatened medicinal plant resources.

## Abbreviations

CM: Chinese medicine; GIS: geographic information system; TCMGIS-I: a GIS-based program for the distribution prediction of traditional Chinese medicine; TCMGIS-II: the improved version of TCMGIS-I program; *C*_*v*_: coefficient of variation; One-way ANOVA: one-way analysis of variance; PCA: principal components analysis; PC: a principal component; SE: standard error.

## Competing interests

The authors declare that they have no competing interests.

## Authors' contributions

SC designed the study and revised the manuscript. HY examined the specimens and wrote the manuscript. CX conducted the TCMGIS-II analysis, JS and YZ helped specimen collection and statistical analysis. All authors revised the manuscript. All authors read and approved the final version of the manuscript.
